# The mechanism of action of paeoniae radix rubra–angelicae sinensis radix drug pair in the treatment of rheumatoid arthritis through PI3K/AKT/NF-κB signaling pathway

**DOI:** 10.3389/fphar.2023.1113810

**Published:** 2023-03-13

**Authors:** Jia Li, Xiaofei Zhang, Dongyan Guo, Yajun Shi, Shihao Zhang, Ruiying Yang, Jiangxue Cheng

**Affiliations:** ^1^ Department of Pharmaceutics, The Key Laboratory of Basic and New Drug Research of Traditional Chinese Medicine, Shaanxi University of Chinese Medicine, Xianyang, China; ^2^ Department of Pharmaceutics, The Key Laboratory of Modern Preparation of Traditional Chinese Medicine, Ministry of Education, Jiangxi University of Chinese Medicine, Nanchang, China

**Keywords:** paeoniae radix rubra-angelicae sinensis radix drug pair, rheumatoid arthritis, UPLC-Q-TOF-MS/MS, network pharmacology, molecular docking, mechanism of action

## Abstract

**Objective:** To investigate the effects and mechanisms of Paeoniae radix rubra–Angelicae sinensis radix (P-A) drug pair in the treatment of rheumatoid arthritis (RA).

**Methods:** Mass spectrometry was employed to accurately characterize the main components of the P-A drug pair. Network pharmacology was used to analyze the main components and pathways of the P-A drug pair in the treatment of RA, and Discovery Studio software was used to molecularly dock the key proteins on the pathway with their corresponding compounds. The levels of serum TNF-a, IL-1β, and IL-6 were measured by enzyme linked immunosorbent assay (ELISA). The histopathology of the ankle joint was observed by hematoxylin-eosin (HE) staining, and the positive expression of p-PI3K, p-IKK, p-NF-κB, and p-AKT in the synovial tissue of the ankle joint was detected by immunohistochemical analysis. Finally, the expression of PI3K, IKK, and AKT and their phosphorylation levels were determined by western blot in each group of rats.

**Results:** Network pharmacology combined with molecular docking analysis revealed that the pharmacodynamic mechanism of the P-A drug pair for the treatment of RA may be related to the contents of caffeic acid, quercetin, paeoniflorin, and baicalein in the regulation of the expression of the PI3K/AKT/NF-κB signaling pathway and the targets of PIK3CA, PIK3R1, AKT1, HSP90AA1 and IKBKB in the pathway. Compared with the model group, the P-A drug pair significantly improved the pathological changes of the synovial tissue and reduced feet swelling in RA model rats. Moreover, it regulated the levels of TNF-α, IL-1β, and IL-6 in serum (*p* < 0.05). The results of the immunohistochemical analysis and western blot showed that the expression of PI3K, IKK, NF-κB, and AKT decreased after phosphorylation in the synovial tissue (*p* < 0.05).

**Conclusion:** The P-A drug pair exhibited an inhibitory effect on the hyperactivation of the PI3K/AKT/NF-κB signaling pathway in the synovial membrane of RA rats. The mechanism may be related to the downregulation of the phosphorylation levels PI3K, IKK, NF-κB, and AKT, which in turn decreased inflammatory cell infiltration and synovial membrane proliferation.

## 1 Introduction

RA is a chronic autoimmune disease that affecting joints, with a global prevalence of about 0.5%–1% (Zhang et al., 2019). It has become the world’s number one disabling disease due to its high risk of deformation and disability, which seriously affects patients’ quality of life (Fang et al., 2020; Roberts et al., 2020; McDonald et al., 2021). The main pathological features of RA are inflammatory changes in synovial tissues, cartilages, and bones, resulting in joint lesions triggered by synovitis, which can cause joint ankylosis and deformity. These pathologies severely impair the function of the joints in advanced stages and eventually lead to disability and death (Ngian, 2010; Alabarse et al., 2018; Scherer et al., 2020). The dampness heat obstruction type is the most common traditional Chinese medicine subtypes of RA and is it more severe. Currently, Western medicine mainly uses anti-inflammatory and anti-rheumatic drugs and biological immunosuppressants to treat RA. These drugs can improve pain symptoms, but they are expensive and cannot effectively control the progression of RA with a single use. In addition, the side effects include serious gastrointestinal reactions, cardiovascular injuries, and other toxic implications (Schnitzer, 2006; Mathews et al., 2016). In contrast, the natural active ingredients from traditional Chinese medicine have the advantages of low toxicity and few adverse effects, making them ideal candidates in the treatment of RA. They can not only effectively relieve the symptoms of RA but delay the progression of the disease to a certain extent. Thus, multi-target therapy *via* traditional Chinese medicine is a better choice (Boleto et al., 2019; Chen et al., 2019; Tasneem et al., 2019).

The P-A drug pair is commonly used in clinical practice to invigorate and tonify blood, and there are 342 blood invigorating and tonifying prescriptions that contain that drug pair in the Traditional Chinese Medicine Prescription Dictionary. Studies have shown that Angelicae sinensis radix has analgesic effects and it can inhibit various acute and chronic inflammatory reactions (Xu and Yuan, 2015). Angelicae sinensis radix-based medicine, Danggui Sini Decoction, can improve the symptoms and pathological progression of RA by affecting the intestinal microbiota and its metabolites (He et al., 2023). Huey-En Tzeng et al. showed that Paeoniae radix rubra has anti-inflammatory effects and it can effectively stimulate osteoclast differentiation in RAW264.7 cells and monocytes (Tzeng et al., 2018). Studies have shown the active components in Paeoniae radix rubra, such as paeoniflorin, can produce anti-inflammatory and immunomodulatory effects by restoring abnormal signal transduction in synovial cells (Zhang and Wei, 2020). In addition, quercetin can inhibit the release of pro-inflammatory mediators (Li et al., 2016; Tabrizi et al., 2020). However, the pharmacological basis and mechanism of action of the P-A drug pair for the treatment of RA remain unclear. In this study, we aimed to successfully replicate the early stage of heat poison and blood stasis syndrome in a rat model, and we have proved that P-A drug pair significantly improved the hemorheology, coagulation function, and inflammatory factor in rats, and the effect was better than that of Paeoniae radix rubra or Angelicae sinensis radix alone (Cheng et al., 2019a; Cheng et al., 2019b). According to previous studies, a collagen-induced arthritis (CIA) model was induced in rats stimulated by rheumatic fever (wind speed 6 m/s, relative humidity 90%, temperature 37°C for 15 d), and the dampness heat obstruction type CIA model rats were successfully prepared. The therapeutic effect of the P-A drug pair on the CIA model rats was preliminarily explored.

**FIGURE 1:**
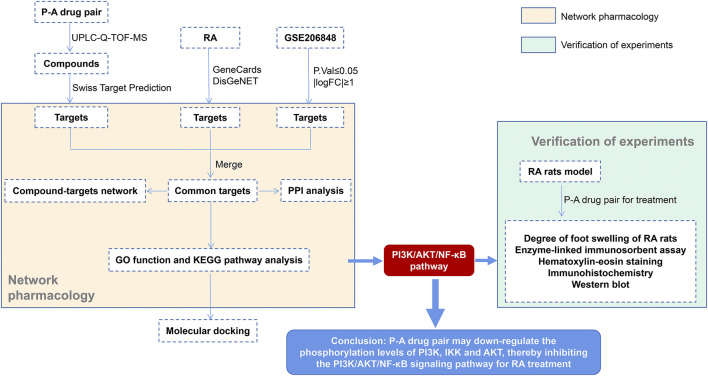
Detailed flowchart of the study.

Ultra-performance liquid chromatography–quadrupole-time of flight-mass spectrometry (UPLC-Q-TOF-MS/MS) technique is a widely used qualitative method in the field of Chinese medicine, combining the efficient chromatographic separation capability of UPLC and the high sensitivity of Q-TOF high-resolution mass spectrometry. This technique can rapidly characterize the complex components of Chinese medicines (Zhang et al., 2020). Network pharmacology provides a new strategy to study the mechanism of action of traditional Chinese medicines by constructing a network to analyze the active component–target action pathways (Niu et al., 2021). In this study, we use the liquid chromatography-mass spectrometry technology, network pharmacological analysis, and *in vitro* experiments to study the mechanism of action of drugs in a more comprehensive manner. The key components in the P-A drug pair were identified by UPLC-Q-TOF-MS/MS, and in combination with the network pharmacology, the target and the mechanism of action of the P-A drug pair for the treatment of RA were predicted. Furthermore, the CIA rat model was established for preliminary verification. The serum levels of TNF-a, IL-1β, and IL-6 were determined by ELISA. The ankle joint histopathology was observed by HE staining. The positive expression of p-PI3K, p-IKK, p-NF-κB, and p-AKT in the synovial tissue of the ankle joint was detected by immunohistochemical, and the expression and phosphorylation levels of PI3K, IKK and AKT were determined by western blot. A flowchart of this study is shown in Figure 1.

## 2 Materials and methods

### 2.1 Materials

Paeoniae radix rubra and Angelicae sinensis radix were purchased from Shaanxi Sciendan Pharmaceutical Co., Ltd. and identified by Professor Cheng Huyin of Shaanxi University of Chinese Medicine. The rat TNF-α ELISA kit (MM-0180R1), rat IL-1β ELISA kit (MM-0047R1), and rat IL-6 ELISA kit (MM-0190R1) purchased from Jiangsu Enzyme Immunoassay Industrial Co.,Ltd. Bovine Type II collagen (L22S11C125306) was purchased from Shanghai Yuanye Bio-Technology Co.,Ltd., and full Frances adjuvant (J04GS149798) was purchased from Shaanxi Shuoyan Chemical Technology Co., Ltd. Antibodies against PI3K (ET1608-70), AKT (ET1609-47), p-AKT (ET1612-73), IKK (ET1611-15) and NF-κB (ET1604-27) were obtained from Hangzhou HuaAn Biotechnology Co.,Ltd., antibodies against p-PI3K (AF3241) was obtained from Affinity Biosciences Group Ltd., antibodies against p-IKK (bs-3232R) was obtained from Beijing Biosynthesis Biotechnology Co.,Ltd.

### 2.2 UPLC-Q-TOF-MS/MS for the determination of the components of the P-A drug pair

#### 2.2.1 Preparation of the test solution

In the treatment of RA, the maximum dose of Paeoniae radix rubra was 60 g, and the maximum dose of Angelicae sinensis radix was 50 g. Combined with the total prescription dosage for the treatment of the dampness heat obstruction type RA, and the common ratio of Angelicae sinensis radix and Paeoniae radix rubra is 1:1, the dosage is 40 g of Angelicae sinensis radix and 40 g of Paeoniae radix rubra (Xing, 2022). According to the conversion coefficient table of 60 kg adult and animal drug dose, the daily dose of rats was calculated to be 7.2 g/kg. Angelicae sinensis radix and Paeoniae radix rubra dried herbs were ground into a coarse powder and mixed well, in the ratio of 1:1. The mixture was placed in a conical flask with a stopper, and 8 volumes of water was added. The mixture was extracted for 0.5 h and refluxed for 1.5 h. The distilled extract was filtered with four layers of gauze to obtain the P-A drug pair decoction. Five milliliters of the decoction was transferred to a 10-mL volumetric flask, and then the same volume of methanol was added into it. The mixture was passed through a 0.22-μm microporous filter membrane, and the filtrate obtained was used as the P-A drug pair decoction for testing.

#### 2.2.2 Preparation of the control solution

The reference standards of chlorogenic acid, caffeic acid, ethyl gallate, 3-butyl-phthalide, benzoyloxypaeoniflorin, senkyunolide I, gallic acid, oxypaeoniflorin, vanillic acid, albiflorin, paeoniflorin, ferulic acid, 1,2,3,4,6-pentagalloylglucose, benzoylpaeoniflorin, paeonol, and ligustilide (the purities of all standards were above 98.0%) were supplied by Shanghai Yuanye Bio-Technology Co., Ltd. Appropriate amounts of the reference standards were mixed and dissolved in methanol in a 10-mL measuring flask, then filtered and set aside.

#### 2.2.3 Chromatographic and mass spectrometric conditions

The determination was performed on Acquity UPLC®BEH C18 column (100 mm × 2.1 mm, 1.7 μm) with mobile phases consisting of 0.1% formic acid acetonitrile (A) −0.1% formic acid aqueous solution (B) in gradient elution (0–1 min, 2 A; 1–5 min, 2%–5% A; 5–9 min, 5%–9.5% A; 9–12 min, 9.5% A; 12–18 min, 9.5%–15% A; 18–25 min, 15%–20% A; 25–30 min, 20%–50% A; 30–37 min, 50%–100% A; 37–38 min, 100% A; 38–40 min, 100%–2% A; 40–42 min, 2% A). The column temperature was 30°C; the flow rate was 0.2 mL/min; and the injection volume was 2 μL.

A triple TOFTM 5600+ time-of-flight liquid mass spectrometer was used for quantitation. Electrospray ion source, positive and negative ion temperature were 600°C and 550°C, spray voltage was 5.5 kV and −4.5 kV respectively. Atomization gas was N2, scanning range was m/z100∼2000, cracking voltage was ±80V, collision energy was ±10 eV, and the total data collection time was 42 min.

### 2.3 Targets of the active compounds of the P-A drug pair and disease targets of rheumatoid arthritis

The targets of the drug were predicted by Swiss TargetPrediction database(http://swisstargetprediction.ch/) (Daina et al., 2019). With “rheumatoid arthritis” as the keyword from the GeneCards database (https://www.genecards.org/) (Stelzer et al., 2016) and DisGeNET database(https://www.disgenet.org/) (Piñero et al., 2021), the related targets of RA were obtained. In this study, we also screened and downloaded GSE206848 microarray data from the GEO database (https://www.ncbi.nlm.nih.gov/geo/) (Barrett et al., 2013), In addition, we obtained samples of normal and RA synovial tissues, and used R software (*p* ≤ 0.05 and |logFC| ≥ 1) to determine differentially expressed genes and to generate volcano plots.

### 2.4 Network construction

The screened active compound targets and RA targets were mapped by Venny 2.1.0(https://bioinfogp.cnb.csic.es/tools/venny/index.html), and the intersection was obtained. The final target was the target of action of the P-A drug pair in RA. Using the STRING platform (https://string-db.org/) (Szklarczyk et al., 2021) with the confidence level set at 0.9, the target–protein interaction relationships for the action of the P-A drug pair in RA were obtained, saved in TSV format, and imported into Cytoscape 3.7.2 for topological analysis. Cytoscape software was used to construct the active compounds of the P-A drug pair and their target networks in RA. Topological analysis was also performed.

### 2.5 Analysis of gene ontology (GO) and kyoto encylopedia of genes and genomes (KEGG)

Based on the core targets screened in the above process, GO and KEGG signaling pathway analysis were performed on the intersecting targets using the cluster Profiler package (Yu et al., 2012) in R 4.0.2 software at *p* < 0.05, and the key signaling pathways for the treatment of RA with the P-A drug pair were explored.

### 2.6 Molecular docking

Molecular docking of key targets of PI3K/AKT/NF-κB pathway with their corresponding components was performed to predict their interactions. The SDF structure files of potential active compounds were downloaded from PubChem (https://pubchem.ncbi.nlm.nih.gov), and the PDB structure files of core targets were downloaded from PDB database (https://www.rcsb.org/) (Burley et al., 2017). The LibDock module in Discovery Studio 4.5 Client software was used for molecular docking, and the docking score value was obtained. The higher the score value, the more stable the ligand-receptor binding.

### 2.7 Animals experiment

#### 2.7.1 Experimental animals

SPF male SD rats, body weight 180–220 g, were purchased from Chengdu Dasuo Laboratory Animal Co., LTD., license number SCXK 2020–030. They were fed at humidity (50 ± 10) % and temperature (25 ± 2)°C and the animal experiment was approved by the Animal Experiment Ethics Committee of Shaanxi University of Chinese Medicine (Approval No. SUCMDL20210930001).

#### 2.7.2 Modeling, grouping, and drug administration

Sixty SPF-grade male SD rats were randomly divided into six groups (*n* = 10): the normal control group, the model group, the Tripterygium glycoside tablet group (20 mg/kg) (Wang et al., 2012), and the low (3.6 g/kg), medium (7.2 g/kg), and high (14.4 g/kg) P-A drug pair dose groups, with 10 animals in each group. Except for the normal group, the remaining 50 only established the CIA model. Under the condition of ice bath, the 2 mg/mL bovine type II collagen solution was mixed with the same volume of Frauchian complete adjuvant by a homogenizer, and the emulsion was fully mixed and emulsified, and the bovine type II collagen emulsion was fully emulsified (emulsification standard: the emulsion was not dispersed when dropped into the water), and the concentration of collagen emulsion was prepared as 1 mg/mL each rat were subcutaneously injected with 0.1 mL of collagen emulsion at the caudal root and two hind paws on the first day for initial immunization. After 7 days, the rats in the experimental groups were repeatedly injected with the same dose of collagen emulsion at the same locations to enhance immunization. After 14 days, the rats showed severe swelling of the hind toes, and their ankle joint diameter increased by ≥ 2 mm. Thus, the CIA model rats were successfully established. At the beginning of 15 days of modeling, the rats were gavaged with the corresponding drugs in the low, medium, and high P-A dose groups, as well as Tripterygium glycoside tablet group once daily for 38 d. The vernier caliper was used to measure the thickness of the posterior plantar.

#### 2.7.3 General observation of rats

The changes in the color of their body hair, mental and activity state, dietary habit, and joint swelling were observed daily.

#### 2.7.4 Serum inflammatory factor level

One hour after the last administration, 5 mL of blood was taken from the abdominal aorta of rats. The blood sample stood for 30 min and then was centrifuged at 3,000 r/min for 15 min to separate the serum. The levels of TNF-α, IL-1β, and IL-6 in the serum were determined by using an ELISA kit, according to the manufacturer’s instructions.

#### 2.7.5 Histopathology of rat ankle joint

The rats were executed by decervicalization, and their left hind feet were taken, fixed with 4% paraformaldehyde, decalcified with 10% EDTA solution, dehydrated by a series of ethanol gradient solutions, treated with xylene, embedded in paraffin, and sectioned. HE staining was used for routine staining. After dehydration, the sections were cleared with xylene and then sealed with neutral gum. The histopathological changes in the ankle joints of the rats were observed under a microscope.

#### 2.7.6 Immunohistochemical

The left ankle joint tissue sections were dewaxed with xylene, and repaired antigen with sodium citrate buffer. After treating with 3% hydrogen peroxide, the sections were rinsed, sealed with 10% goat serum, and p-PI3K, p-IKK, p-NF-κB and p-AKT antibodies were added dropwise. The tissue sections were incubated overnight at 4°C. Biotin-labeled secondary antibodies were added for incubation, and the color developed by dropwise addition of diaminobenzidine chromogenic solution was observed. Hematoxylin was re-stained, dehydrated, cleared, sealed, and photographed. Quantitative analysis of the positive proteins was performed by using Image-Pro plus software.

#### 2.7.7 Western blotting

Protein was extracted from the synovial tissue of the right ankle of the rats, and the bicinchoninic acid method was used for protein quantification. Briefly, 9 µL of the protein sample was separated by 10% SDS-PAGE and closed with 5% skim milk for 1 h. The primary antibody was added and incubated overnight, then the secondary antibody was added for incubation. An enhanced chemiluminescence kit was used for color development, and protein bands were photographed in a gel imager system. The relative protein expression (with β-actin as the internal reference protein) was analyzed.

### 2.8 Statistical methods

All data are expressed as the mean ± standard deviation, and Graph Pad Prism 9.0.0 software was used to process the data. One-way ANOVA and Tukey’s multiple comparisons tests were used to compare the data. *p* < 0.05 indicated that the difference was statistically significant.

## 3 Results

### 3.1 UPLC-Q-TOF-MS/MS assay results

**FIGURE 2:**
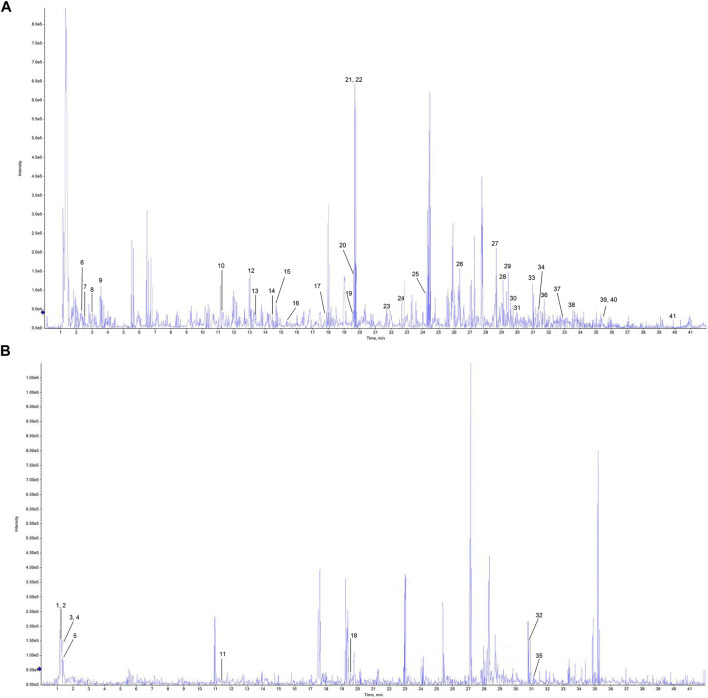
Total ion flow chromatogram of the P-A drug pair. **(A)** Negative ion diagram. **(B)** Positive ion diagram.

The detected data were imported into Peak View 2.2 software, and NIST 2017, NIST E&L_HR-MS/MS_1.0, TCM Library 1.0, TCM MS/MS Library were used as the matching library of P-A drug pair. Combined with the mass spectrum information of each compound, comparison with the reference standards, reference to the relevant literature data, and compounds in the P-A drug pair were identified and classified. A total of 41 compounds were identified, among which 19 components were found in Paeoniae radix rubra, 19 components identified in Angelicae sinensis radix, and there were three compounds common to both herbs. Among them, compounds 9, 12, 14, 15, 19, 20, 21, 22, 23, 26, 28, 29, 32, 33, 39, 40 were identified as gallic acid, chlorogenic acid, caffeic acid, vanillic acid, ethyl gallate, oxypaeoniflorin, paeoniflorin, albiflorin, ferulic Acid, 1,2,3,4, 6-pentagalloylglucose, paeonol, benzoyloxypaeoniflorin, senkyunolide I, benzoylpaeoniflorin, ligustilide, 3-butyl-phthalide, respectively, by comparison with reference standards. The identification results are summarized in Table 1, and the total ion flow diagram of the P-A drug pair is shown in Figure 2.

**TABLE 1:** Identification of the chemical components of the P-A drug pair.

No	RT (min)	Identification	Formula	PPM	Calculated mass	Measured mass	Selected ion	Fragmentation	Source
1	1.25	Histidine Zhu et al. (2022)	C6H9N3O2	4.5	156.0767	156.0774	[M + H]^+^	110.0710, 156.0782 Zhu et al., (2022)	A
2	1.25	L(+)-Arginine Zhu et al. (2022)	C6H14N4O2	0.5	175.1044	175.1045	[M + H]^+^	116.0747,175.1296 Zhu et al. (2022)	A
3	1.27	Arbutin Wu et al. (2021)	C12H16O7	−3.4	273.08232	273.08139	[M + H]^+^	123.0469 Hu et al. (2022)	P
4	1.32	Glutamic acid Zhu et al. (2022)	C5H9NO4	0.4	148.04589	148.04594	[M + H]^+^	84.0446, 102.0521, 130.0543 Zhu et al. (2022)	A
5	1.38	Proline Dong and Chen (2016)	C5H9NO2	0	116.05606	116.05606	[M + H]^+^	70.0643 Li et al. (2022)	A
6	2.3	Adenosine Zhu et al. (2022)	C10H13N5O4	−0.2	266.08946	266.08941	[M-H]^-^	134.0467 Zhu et al. (2022)	A
7	2.62	Amber acid Ma et al. (2022)	C4H6O4	0.8	117.01933	117.01943	[M-H]^-^	117.0229 Liu et al. (2022a)	A
8	3.03	Guanosine Zhu et al. (2022)	C10H13N5O5	−0.1	282.0844	282.08438	[M-H]^-^	150.0422, 133.0152, 282.0842 Zhu et al. (2022)	A
9^a^	3.62	Gallic acid Zou et al. (2022)	C7H6O5	1.1	169.01425	169.01443	[M-H]^-^	169.0142, 125.0242 Zhu et al. (2022)	P
10	11.33	L-Tryptophan Ma et al. (2022)	C11H12N2O2	1.8	203.0826	203.08296	[M-H]^-^	116.0510, 142.0665, 203.0835 Liu et al. (2022a)	A
11	11.37	Propyl gallate Ruan et al. (2003)	C10H12O5	6.2	213.0757	213.077	[M + H]^+^	107.0876 Hu et al. (2022)	P
12^a^	13.08	Chlorogenic acid Zhu et al. (2022); Zou et al. (2022)	C16H18O9	−1.1	353.08782	353.08741	[M-H]^-^	135.0513, 173.0432, 191.0564 Liu et al. (2022a)	P-A
13	13.29	Catechin Zou et al., (2022)	C15H14O6	0.3	289.07176	289.07185	[M-H]^-^	109.0304, 123.0458, 137.0229, 245.0824 Yang et al. (2023)	P
14^a^	14.56	Caffeic acid Zhu et al. (2022)	C9H8O4	1.4	179.03498	179.03523	[M-H]^-^	135.0454 Zhu et al. (2022)	A
15^a^	14.69	Vanillic acid Liu et al. (2017); Wu et al. (2021)	C8H8O4	1.6	167.03498	167.03524	[M-H]^-^	167.0379, 123.0455 Liu et al. (2022a)	P-A
16	15.33	Protocatechuic acid Liu et al., (2017); Liu et al. (2022b)	C7H6O4	1.2	153.01933	153.01952	[M-H]^-^	109.0291 Li et al. (2022)	P-A
17	17.88	Fraxetin Zou et al. (2022)	C10H8O5	3.6	207.02989	207.03063	[M-H]^-^	207.1382, 192.0056 Zou et al. (2022)	P
18	19.62	7-Hydroxycoumarin Liu et al. (2022c)	C9H6O3	3	163.0389	163.0394	[M + H]^+^	117.0368, 135.0482, 163.0424 Liu et al. (2022c)	A
19^a^	19.69	Ethyl gallate Zou et al. (2022)	C9H10O5	0.6	197.04555	197.04568	[M-H]^-^	169.0129, 124.0178, 125.0238 Liu et al. (2022b)	P
20^a^	19.74	Oxypaeoniflorin Zou et al. (2022)	C23H28O12	−1.3	495.1508	495.15017	[M-H]^-^	137.0245, 165.0555, 465.1396 Liu et al. (2022b)	P
21^a^	19.75	Paeoniflorin Zou et al. (2022)	C23H28O11.HCOOH	−1.5	525.16136	525.16059	[M + HCOO]^-^	121.0300, 165.0579, 327.1095 Liu et al. (2022b)	P
22^a^	19.75	Albiflorin Hu et al. (2022)	C23H28O11	−1.9	479.15587	479.15496	[M-H]^-^	121.0307, 479.1563 Liu et al. (2022b)	P
23^a^	21.73	Ferulic acid Ruan et al. (2003)	C10H10O4	1.8	193.05063	193.05097	[M-H]^-^	178.0275, 149.0607, 134.0374 Liu et al. (2022b)	A
24	22.74	Ellagic acid Liu et al. (2017)	C14H6O8	−0.7	300.99898	300.99878	[M-H]^-^	283.9981, 245.0091, 229.0142 Yang et al. (2023)	P
25	24.23	Benzoic acid Wu et al. (2021)	C7H6O2	1.7	121.0295	121.0297	[M-H]^-^	121.0321 Yang et al. (2023)	A
26^a^	26.32	1,2,3,4,6-Pentagalloylglucose Zhu et al. (2022)	C41H32O26	−1	939.11089	939.10998	[M-H]^-^	769.095 Yang et al. (2023)	P
27	28.72	3,5-Di-O-caffeoylquinic acid Zhu et al. (2022)	C25H24O12	0.4	515.11949	515.11967	[M-H]^-^	191.0568, 353.0872, 515.1222 Zhu et al. (2022)	A
28^a^	29.32	Paeonol Zou et al. (2022)	C9H10O3	1.2	165.05571	165.05591	[M-H]^-^	165.0611 Yang et al. (2023)	P
29^a^	29.65	Benzoyloxypaeoniflorin Liu et al. (2022b)	C30H32O13	−0.4	599.17699	599.17675	[M-H]^-^	137.0256, 121.0326 Yang et al. (2023)	P
30	29.74	Ethyl caffeate Zhu (2018)	C11H12O4	0.7	207.06629	277.06648	[M-H]^-^	207.0649 Zou et al. (2022)	A
31	30.5	Quercetin Li et al. (2022)	C15H10O7	−0.4	301.03539	301.03526	[M-H]^-^	301.1855, 239.0387 Zhu et al. (2022)	P
32^a^	30.83	Senkyunolide I Zou et al. (2022)	C12H16O4	7.5	225.1121	225.1138	[M + H]^+^	161.0940, 165.0513, 179.1040, 189.0901, 207.1020 Li et al. (2022)	A
33^a^	31.01	Benzoylpaeoniflorin Zou et al. (2022)	C30H32O12	−0.7	583.18212	583.18168	[M-H]^-^	583.1844, 553.1727, 431.1323, 165.0554, 121.0303 Yang et al. (2023)	P
34	31.49	Naringenin Wu et al. (2021)	C15H12O5	0.8	271.0612	271.06141	[M-H]^-^	151.0037, 119.0510 Liu et al. (2022a)	P
35	31.49	Baicalein You et al. (2020)	C15H10O5	4.8	271.0601	271.0614	[M + H]^+^	253.0498 Zhu et al. (2022)	P
36	31.73	Isorhamnetin Li et al. (2022)	C16H12O7	−0.7	315.05101	315.05079	[M-H]^-^	151.0024, 300.0253 Li et al. (2022)	P
37	32.99	Senkyunolide F Yang et al. (2022)	C12H14O3	2.7	205.08702	205.08758	[M-H]^-^	161.0962, 205.0865 Yang et al. (2022)	A
38	33.53	Senkyunolide C Zhu et al. (2022)	C12H12O3	2.5	203.07137	203.07188	[M-H]^-^	145.0296, 173.0247, 203.0718 Zhu et al. (2022)	A
39^a^	35.4	Ligustilide Zhu et al. (2022)	C12H14O2	1.3	189.0921	189.09235	[M-H]^-^	161.0917, 189.0991 Zhu et al. (2022)	A
40^a^	35.4	3-Butyl-phthalide Zhu et al. (2022)	C12H14O2	1.3	189.0921	189.0924	[M-H]^-^	143.0464, 161.0970, 171.0769, 189.0940 Zhu et al. (2022)	A
41	39.92	Linoleic acid Liu et al. (2022c)	C18H32O2	−0.1	279.23296	279.23293	[M-H]^-^	279.2335 Liu et al. (2022c)	A

Notes: a, the compounds confirmed by comparison with the reference standards; P, paeoniae radix rubra; A, angelicae sinensis radix; P-A, Paeoniae radix rubra–Angelicae sinensis radix.

### 3.2 Component target acquisition and mapping analysis with RA targets

**FIGURE 3:**
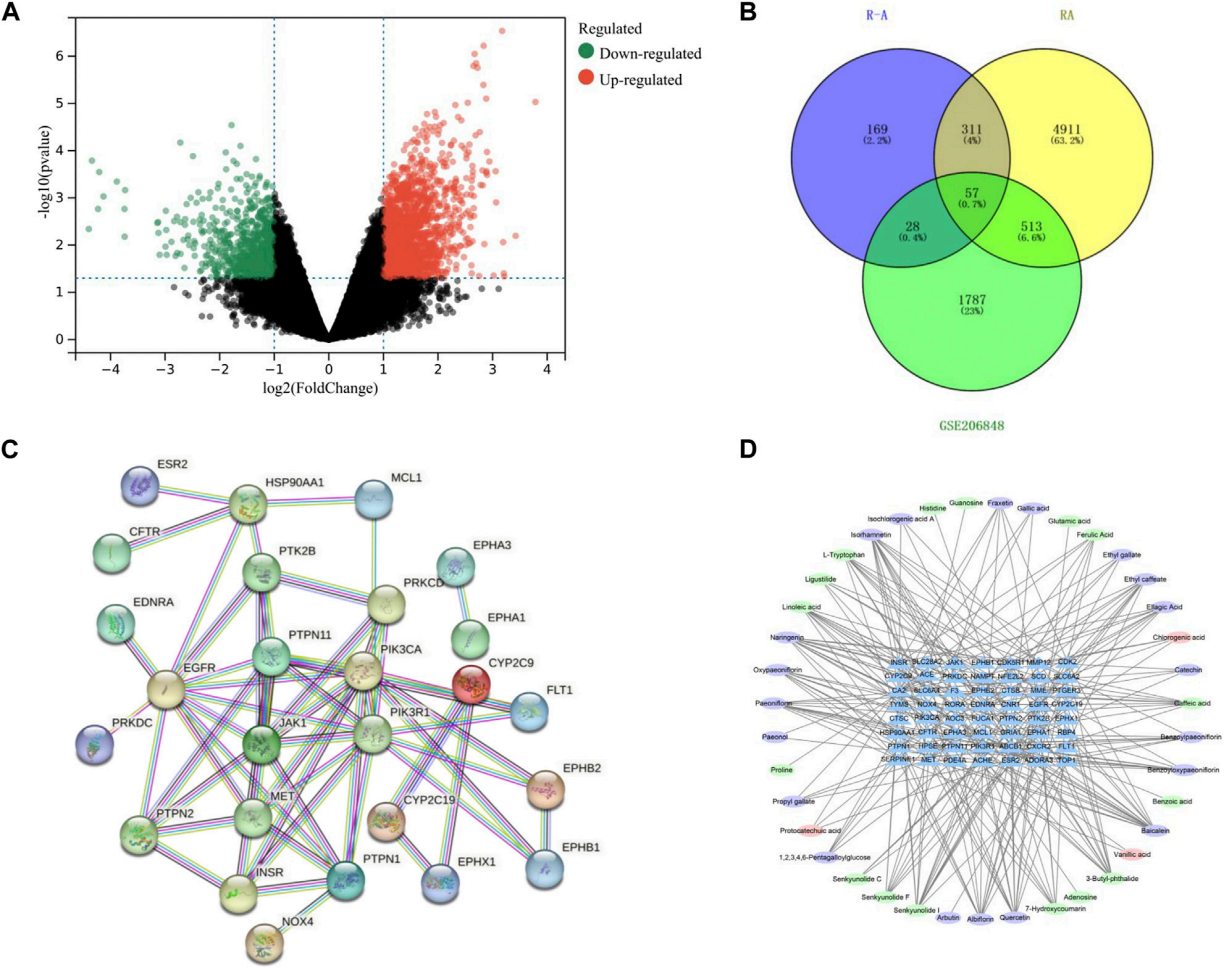
**(A)** Volcano plot of differential genes on GSE206848 chip (no differential genes are shown in black, the gene is upregulated and shown in red. The gene is downregulated and shown in green). **(B)** Venn diagram of the active ingredients in the P-A drug pair and RA target. **(C)** PPI network. **(D)** Network diagram of active ingredient–target pairs of the P-A drug pair (oval represents chemical compositions, and triangle represents targets).

A total of 1,926 component targets were predicted by using Swiss Target Prediction database, and a total of 565 active component targets of the P-A drug pair were obtained after de-weighting. After combining the results of the GeneCards and the DisGeNET databases, a total of 5,792 RA-related targets were obtained after de-duplication. GSE206848 was filtered and normalized by using R software and related software packages to screen for differential genes, and a total of 2,805 differential genes were obtained for upregulated (1,975) and downregulated (830) RA. The volcano map of the differential genes is shown in Figure 3A. Using the Venny 2.1.0 online platform to obtain the common targets of the active ingredient targets of P-A drug pair and RA diseases, 57 intersection targets were finally obtained, as shown in Figure 3B. There were 37 upregulated genes (CFTR, ABCB1, EGFR, HSP90AA1, INSR, GRIA1, PDE4A, SLC28A2, EPHB1, TOP1, NOX4, CYP2C9, PTPN2, PIK3CA, SLC6A4, CDK2, PIK3R1, EPHA3, MCL1, CDK5R1, JAK1, NFE2L2, F3, PTPN11, ESR2, PRKDC, CA2, RORA, CNR1, MET, PTPN1, NAMPT, FLT1, EDNRA, EPHA1, CYP2C19, SERPINE1) and 20 downregulated genes (SCD, TYMS, SLC6A2, PTGER3, RBP4, EPHX1, ADORA3, AOC3, MME, MMP12, ACHE, HPSE, FUCA1, ACE, EPHB2, CTSB, CXCR2, CTSC, PTK2B, PRKCD).

### 3.3 Network construction and analysis

The 57 RA targets of the P-A drug pair were imported into STRING database for analysis, and the confidence level was set to >0.9 to eliminate the isolated target proteins and obtain the protein interaction information. The network was imported into Cytoscape 3.7.2 software for visualization, and network topology analysis was conducted to obtain the PPI of the targets (57 nodes and 56 edges). As shown in Figure 3C, and according to the Degree value, the core targets of P-A drug pair for RA treatment were mainly PIK3R1, EGFR, PIK3CA, PTPN11, JAK1, PTPN1, MET, and HSP90AA1. The compound–target network was mapped by Cytoscape 3.7.2, as shown in Figure 3D, with 95 nodes (38 compounds and 57 targets) and 255 interacting edges. The Degree value was used to screen the main components of P-A drug pair in the treatment of RA. The topological analysis yielded the key compounds baicalein, naringenin, senkyunolide I, quercetin, isorhamnetin, albiflorin, paeoniflorin, 3-butyl-phthalide, and caffeic acid.

### 3.4 GO and KEGG enrichment analysis

**FIGURE 4:**
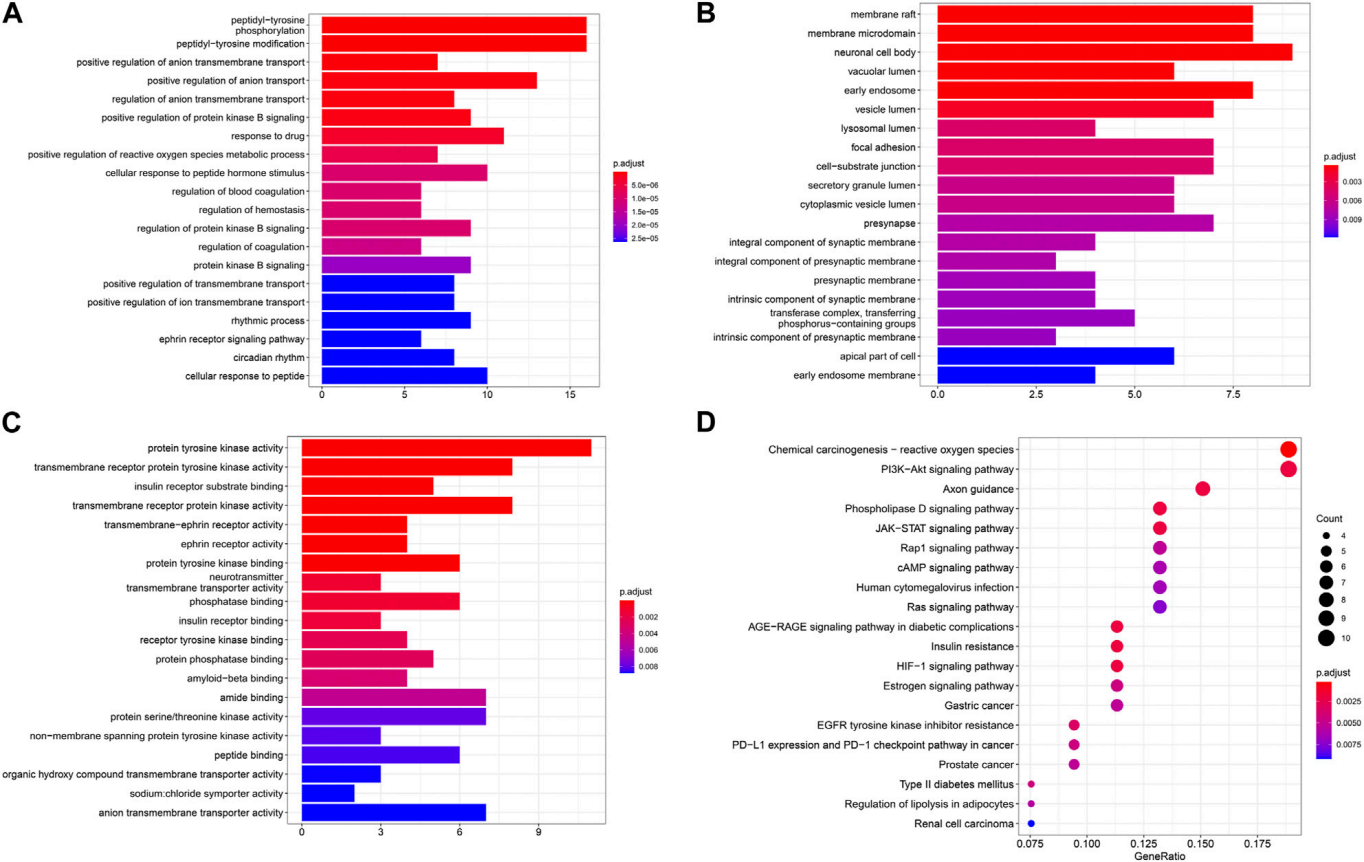
**(A-D)** Results of GO-BP, GO-CC, GO-MF, and KEGG pathway enrichment analysis.

GO and KEGG enrichment analysis of intersection targets was performed by using R 4.0.2 software. At the significance level of *p* < 0.05, 680 related biological processes (BPs), 44 related cell compositions (CCs), and 56 related molecular functions (MFs) were screened (Figures 4A–C). Through KEGG enrichment analysis, a total of 67 signaling pathways were selected according to *p* < 0.05, including PI3K-AKT, JAK-STAT, Rap1, and other signaling pathways, as shown in Figure 4D.

### 3.5 Molecular docking prediction

**FIGURE 5:**
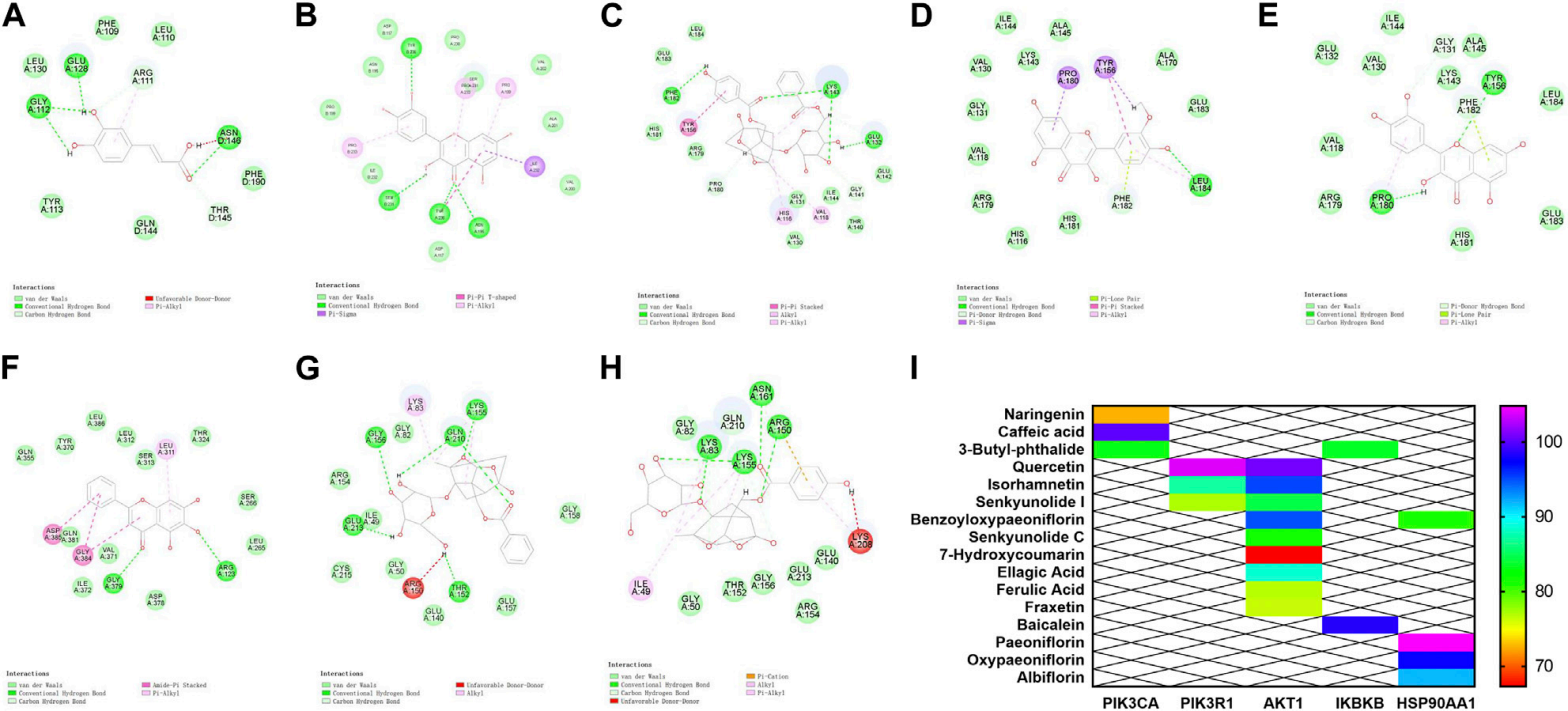
Molecular docking results. **(A)** PIK3CA and caffeic acid. **(B)** PIK3R1 and quercetin. **(C–E)** AKT1 target and benzoyloxypaeoniflorin, isorhamnetin, and quercetin. **(F)** IKBKB target and baicalein. **(G)** HSP90AA1 and paeoniflorin. **(H)** HSP90AA1 and oxypaeoniflorin. **(I)** Heat map of molecular docking score.

The core targets PIK3R1, PIK3CA, AKT1, HSP90AA1 and IKBKB and their corresponding components on the PI3K/AKT/NF-κB pathway were predicted by molecular docking. The core targets were used as molecular dockingreceptors, and the active ingredients corresponding to the core targets were used as molecular docking ligands. LibDock was used for molecular docking. The docking patterns of some compounds and targets are shown in Figures 5A–I, and the molecular docking score heat map is shown in Figure 5J. According to the heat map, the core targets PIK3R1 and quercetin, PIK3CA and caffeic acid, AKT1 and quercetin, HSP90AA1 and paeoniflorin, and IKBKB and baicalein had the best intermolecular affinity.

### 3.6 P-A drug pair treatment alleviates the symptoms associated with RA rats

**FIGURE 6:**
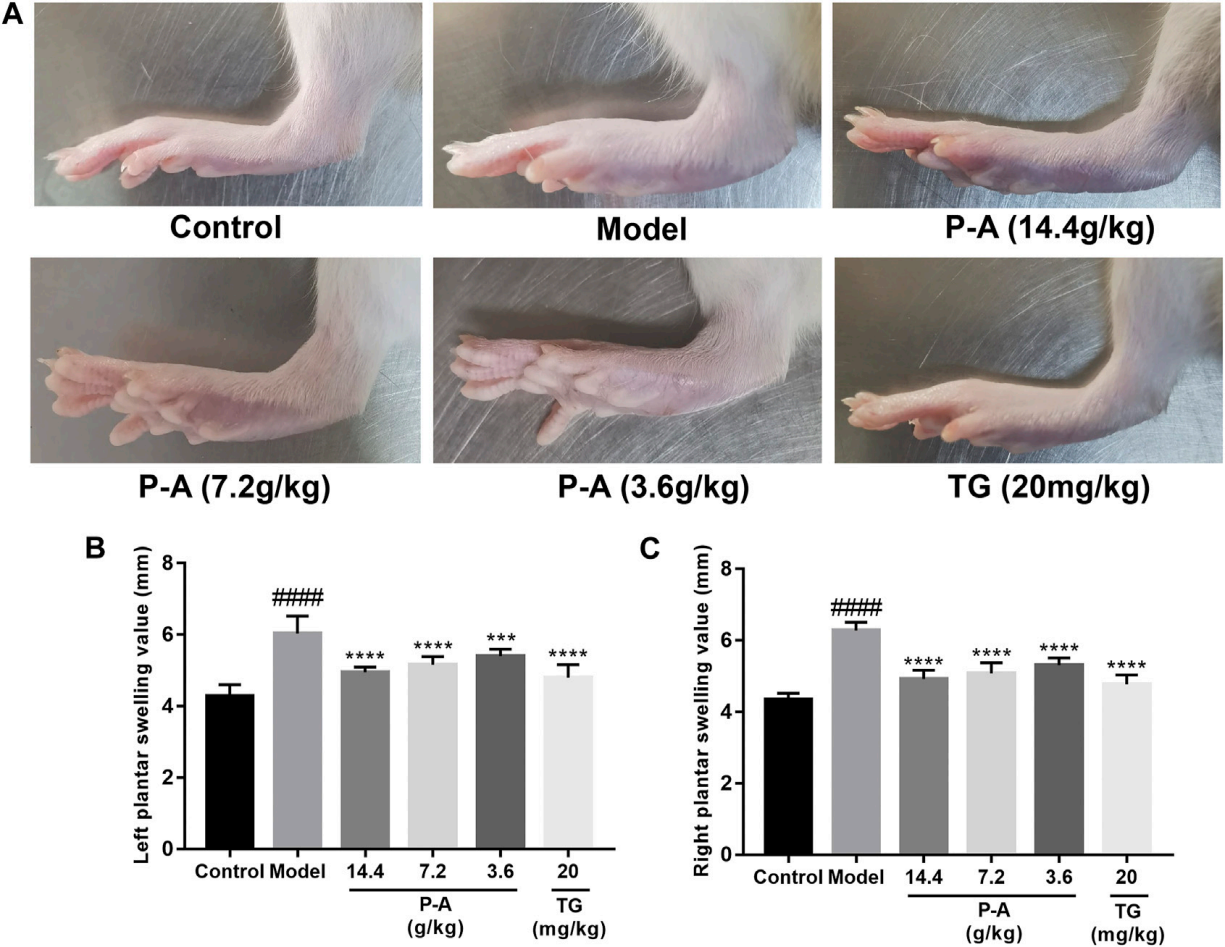
**(A)** Pathological phenotypic changes. **(B)** Left plantar swelling value. **(C)** Right plantar swelling value.

After the second immunization, the rats showed obvious arthritic features, poor mental status, decreased diet, body mass, decreased mobility, as well as obvious redness and deformation of the feet and limbs. The swelling of the feet of the rats in the Tripterygium glycoside tablet group and high-dose P-A drug pair group was significantly reduced (Figure 6).

### 3.7 Contents of TNF-α, IL-1β, and IL-6 in the serum

**FIGURE 7:**
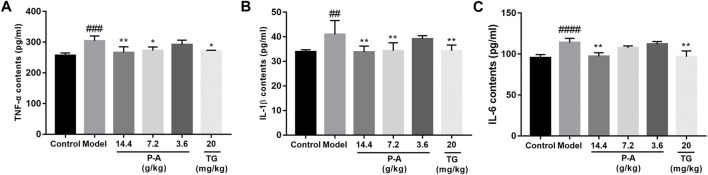
Effects of the P-A drug pair on TNF-α, IL-1β, and IL-6 levels in the serum of RA rats. **(A)** Effect of the P-A drug pair on TNF-α contents. **(B)** Effect of the P-A drug pair on IL-1β contents. **(C)** Effect of the P-A drug pair on IL-6 contents. The data represent the mean ± SD. #*p* < 0.5, ##*p* < 0.01, ###*p* < 0.001, ####*p* < 0.0001 vs. control group; *p < 0.5, **p < 0.01, ***p < 0.001, ****p < 0.0001 vs. model group.

Compared with the normal control group, the levels of TNF-α, IL-1β, and IL-6 in the serum and ankle synovial tissue of rats in the model group increased (*p* < 0.01). Compared with the model group, the levels of TNF-α, IL-1β, and IL-6 in the serum of rats in the high-dose P-A drug pair group decreased (*p* < 0.01), as shown in Figure 7.

### 3.8 Effect of P-A drug pair on histopathological changes

**FIGURE 8:**
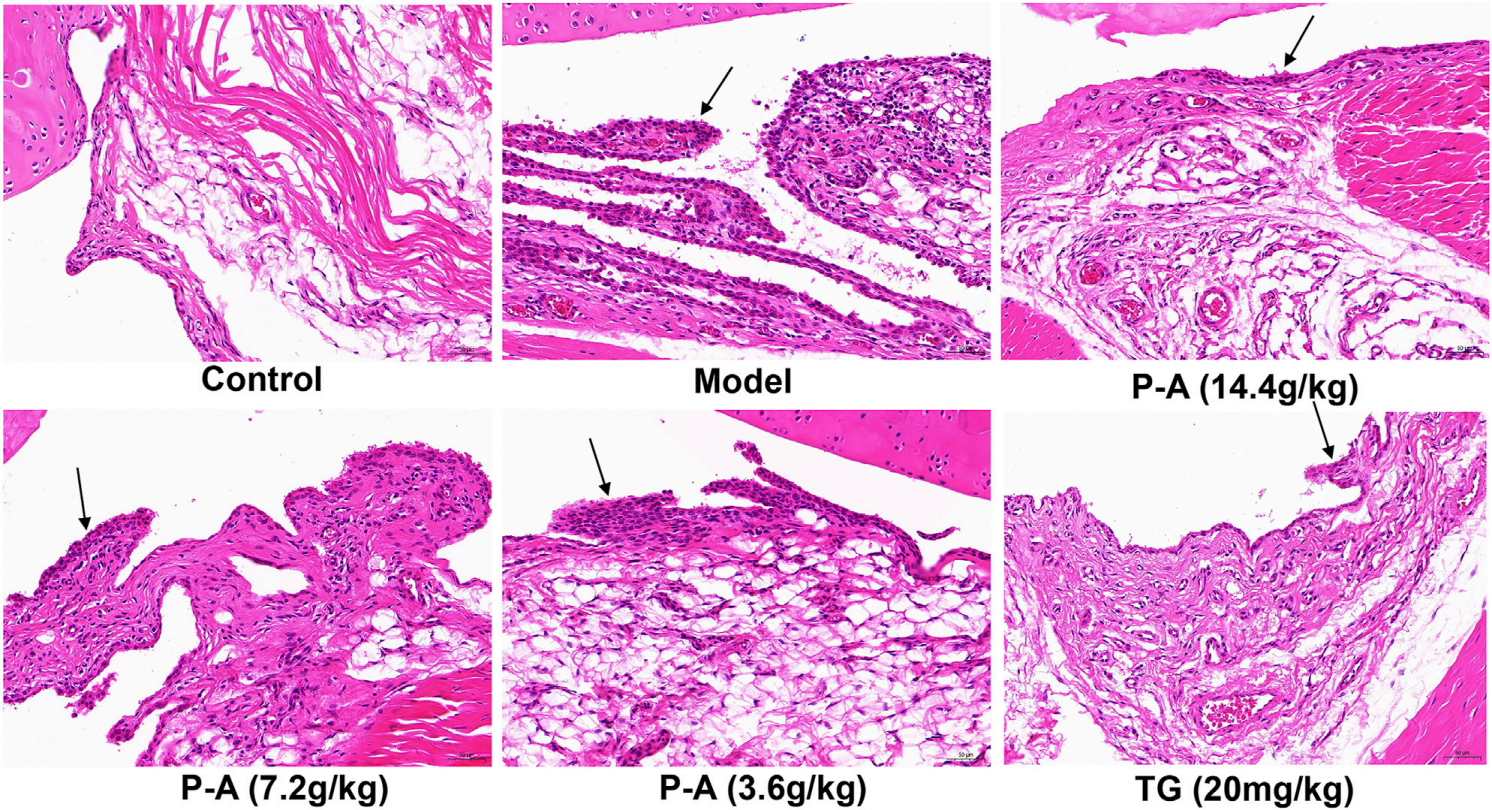
Effect of the P-A drug pair on pathological changes in the synovium of the ankle joint in rats (HE, x 200). Black arrow represents synovial hyperplasia.

As shown in Figure 8, HE staining showed abnormal hyperplasia, disordered arrangement, and infiltration of a large number of inflammatory cells in the ankle synovium of rats in the model group, and the synovial layer was significantly thicker. The synovial membrane of the ankle joint of rats in the high-dose P-A drug pair group was notably recovered, and the inflammatory cell infiltration was reduced.

### 3.9 Determination of p-PI3K, p-IKK, p-NF-κB, and p-AKT protein expression in the synovial tissue of the ankle joint of RA rats by immunohistochemical analysis

**FIGURE 9:**
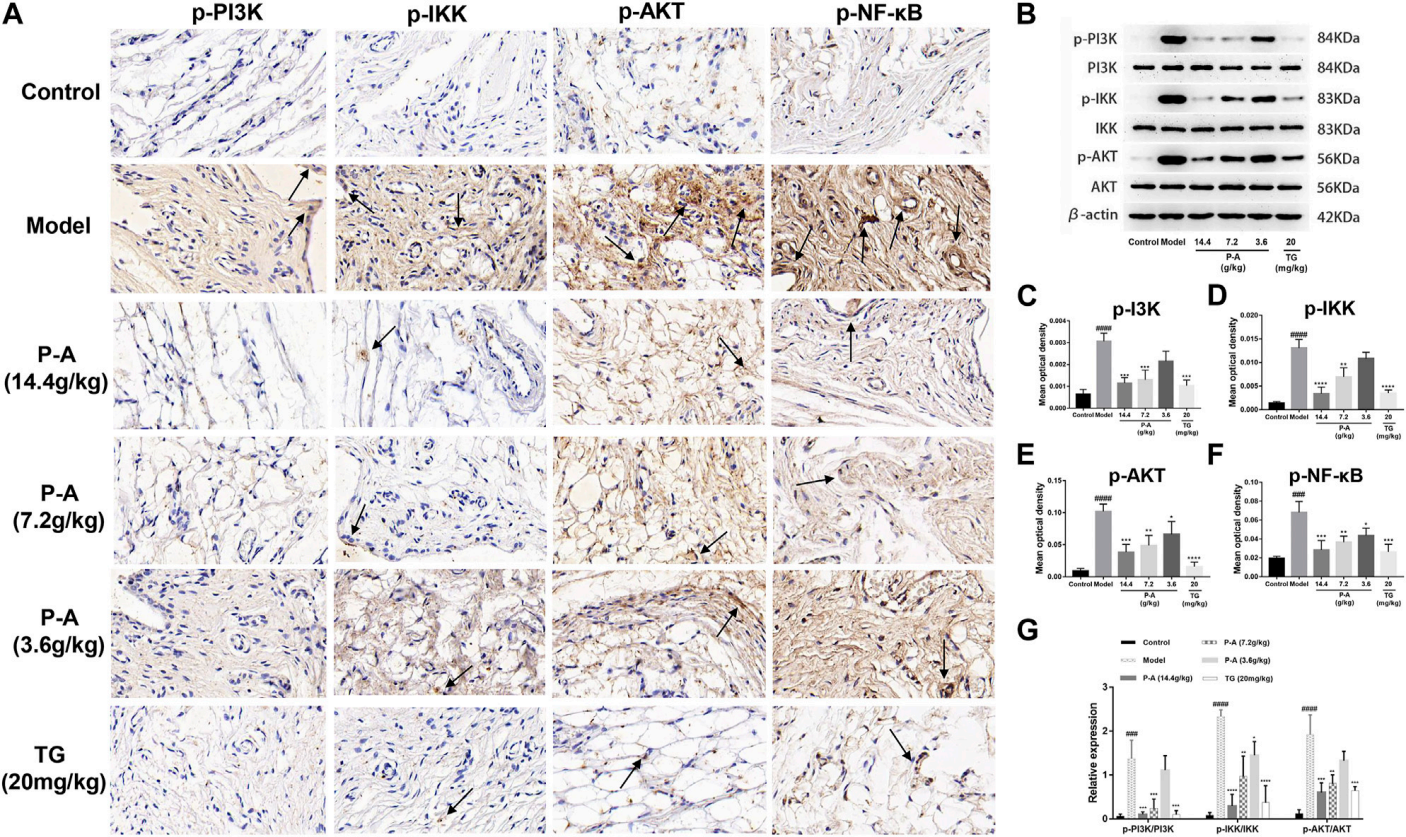
**(A)** Distribution of p-PI3K, p-IKK, p-NF-κB, and p-AKT positive cells in the synovial tissue of the ankle joint in each group (immunohistochemical method, ×100). **(B)** Effect of the P-A drug pair on PI3K, IKK, and AKT protein expression and their phosphorylation levels. **(C–F)** Quantitative analysis of p-PI3K, p-IKK, p-AKT and p-NF-κB. **(G)** Quantitative analysis of *p* PI3K/PI3K, p-IKK/IKK and p-AKT/AKT. The data represent the mean ± SD. #*p* < 0.5, ##*p* < 0.01, ###*p* < 0.001, ####*p* < 0.0001 vs. control group; *p < 0.5, **p < 0.01, ***p < 0.001, ****p < 0.0001 vs. model group.

As shown in Figure 9, in the model group, p-PI3K, p-IKK, p-NF-κB, and p-AKT protein were expressed more than in the control group (*p* < 0.0001), indicating that there was inflammatory injury in the ankle synovial tissue of rats in the model group. The P-A drug pair in the high and medium dose groups, p-PI3K, p-IKK, p-NF-κB, and p-AKT protein expression in ankle tissue decreased (*p* < 0.01), indicating that the expression of inflammatory factors in RA rats could be inhibited by the P-A drug pair.

### 3.10 Expression of PI3K, IKK, and AKT proteins and their phosphorylation levels in the synovial tissue of the ankle joint of RA rats by western blot analysis

As shown in Figure 9, the expression of p-PI3K/PI3K, p-IKK/IKK, and p-AKT/AKT in the model group rats after modeling was significantly increased, compared with the control group (*p* < 0.001). The expression of p-PI3K/PI3K, p-IKK/IKK, and p-AKT/AKT was inhibited to varying degrees by the P-A drug pair in the high and medium dose groups, with significant differences compared with the model group (*p* < 0.01).

### 3.11 Description of the mechanism

**FIGURE 10:**
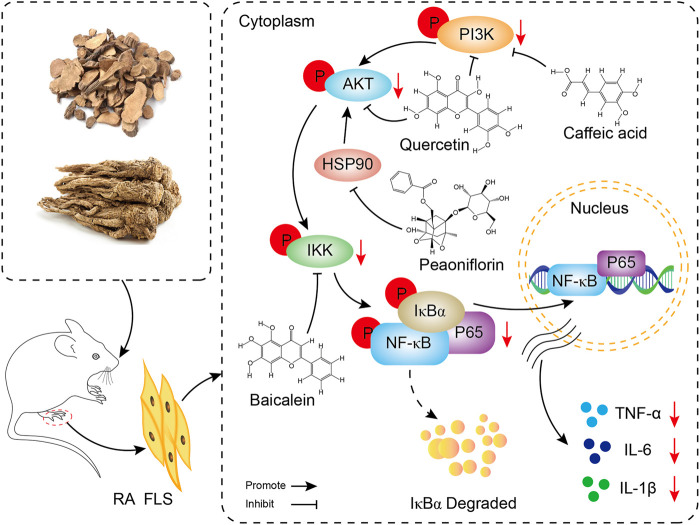
Mechanism display of P-A drug pair in the treatment of RA through PI3K/AKT/NF-κB signaling pathway.

The core targets PIK3R1, PIK3CA, AKT1, HSP90AA1 and IKBKB and their corresponding components in the PI3K/AKT/NF-κB pathway were docked by molecular docking technology. PIK3R1 and quercetin, PIK3CA and caffeic acid, AKT1 and quercetin, HSP90AA1 and paeoniflorin, and IKBKB and baicalin showed the best intermolecular affinity. The rat model of CIA was established by injecting bovine type II collagen emulsion subcutaneously, at the root of the tail and in the paw of the two hind feet. After 38 days of continuous gavage with the P-A drug pair, serum TNF-a, IL-1β, IL-6 levels were measured in each group by ELISA. Ankle joint histopathology was observed by HE staining, and the p-PI3K, p-IKK, p-NF-κB, and p-AKT protein positive expression in the ankle synovial tissues of each group was detected by immunohistochemical analysis. PI3K, IKK, and AKT protein expression and their phosphorylation levels in the ankle synovial tissue of each group were determined by western blot. Combining the results of each experiment, we believe that the mechanism of action of the P-A drug pair in the treatment of RA may be related to the action of some key compounds, such as caffeic acid, quercetin, paeoniflorin, and baicalein, on key targets, including PIK3R1, PIK3CA, AKT1, HSP90AA1, and IKBKB, to downregulate the phosphorylation levels of PI3K, IKK, and AKT. This, in turn, inhibits the release of inflammatory mediators TNF-α, IL-1β, and IL-6 and suppresses the overactivation of the PI3K/AKT/NF-κB signaling pathway in the synovial membrane of RA rats (Figure 10).

## 4 Discussion

RA is a chronic, progressive, and aggressive autoimmune disease with synovitis and extra-articular lesions as the main clinical manifestations (Zhang et al., 2013). Current treatment is mainly focused on reducing joint swelling and pain, controlling the development of arthritis, preventing and reducing joint destruction, and promoting the repair of damaged joints and bone (Wang, 2007). The clinical symptoms and pathological manifestations of the collagen-induced arthritis rat model are very similar to those of RA, making it ideal for studying RA. In the present study, after the establishment of the RA rat model by type II collagen induction, the rats in the model group showed lethargy, loss of appetite, obvious redness and swelling of the limbs, significantly lower body mass, and significantly higher feet swelling, compared with the normal control rats. In addition, hyperplasia of the ankle synovial tissue was observed, with obvious pathological changes. The expression levels of PI3K, IKK, and AKT protein phosphorylation in the model group increased, suggesting that the modeling of RA rats was successful. After the treatment with the P-A drug pair, the swelling of the feet was significantly reduced, the structure of the ankle synovial tissue tended to be normal, and the expression levels of PI3K, IKK, and AKT proteins in the ankle synovial tissue were reduced. These changes indicate that the P-A drug pair is effective in treating RA.

Excluding signaling pathways unrelated to the disease, the results of KEGG enrichment analysis showed that the drug treated RA by acting on PI3K-AKT, JAK-STAT, HIF-1, and other signaling pathways. The PI3K-Akt signaling pathway can regulate the release of inflammatory factors and the formation of enzymes related to proliferation, apoptosis, and inflammation to participate in the pathological process of RA. The PI3K-AKT signaling pathway was found to be widely present and aberrantly activated in RA synoviocytes (Harris et al., 2009). Inhibition of the expression of the PI3K-AKT signaling pathway or anti-apoptotic molecules can induce apoptosis in fibroblast-like synoviocytes, which is therapeutic for RA (Liu and Pope, 2003). PI3K/AKT phosphorylation can activate IL-1β to induce the expression of pro-inflammatory factor TNF-α, IL-6 expression, and the development of inflammation in rat joints (Jiang et al., 2020). In the physiological state, NFκB dimers are bound to their inhibitor IκB and are present in the cytoplasm in a non-activated form. NFκB is mainly localized in the nucleus of synoviocytes, and AKT phosphorylates activates IκB kinase (IKKα), leading to the degradation of IκB, an inhibitor of NFκB. This results in the release of the transcription factor NFκB from the cytoplasm for nuclear translocation and initiation of its target gene expression, thereby promoting cell survival (He et al., 2013). HSP90AA1 can block the nuclear factor κB pathway and reduce the level of inflammatory mediators (Guo et al., 2016). Studies have found that downregulation of the JAK1/STAT3 pathway can reduce proinflammatory cytokines, MMPs inhibition, and inflammatory cell apoptosis, thereby alleviating the clinical symptoms of RA (Wang et al., 2018). HIF-1 plays a role in inflammatory/innate immune responses (Sumbayev and Nicholas, 2010), and activation of the HIF-1 pathway causes downregulation of NF-κB, pro-inflammatory cytokines and plays a protective role in inflammation (Hirai et al., 2018). Therefore, the mechanism of P-A drug pair in the treatment of RA is related to the above inflammation-related pathways.

According to the above analysis results, in this study, the key proteins PIK3R1, PIK3CA, AKT1, HSP90AA1, and IKBKB and their corresponding components in the PI3K/AKT/NF-κB pathway were interlinked by molecular docking technology, and the results indicated that the binding stability between all the active compounds and the key targets was strong. In particular, quercetin, caffeic acid, paeoniflorin, and baicalin scored higher than the other compounds with the key targets. The results suggest that these four compounds are the active ingredients of the P-A drug pair for the treatment of RA. A previous study showed that quercetin reduced joint swelling and inflammation in mice with arthritis and alleviated joint damage (El-Said et al., 2022). By inhibiting the phosphorylation of IκB and IκB kinase, caffeic acid can inhibit the infiltration and secretion of inflammatory cells in joint synovium and play an anti-inflammatory and preventive role (Wang et al., 2017; Nouri et al., 2022). Baicalin can reduce the effect of IL-1β and TNF-α on RA fibroblast-like synovial cells (Humby et al., 2017). Paeoniflorin can reduce joint swelling and subcutaneous hematoma in CIA rats, decrease the mean arthritis index, and reduce the degree of bone destruction (Wu et al., 2014). These activities confirmed the effectiveness of these compounds in the treatment of RA. In this study, the key targets of PI3K/AKT/NF-κB pathway were verified in collagen-induced arthritis animal model. The results showed that P-A drug pair exerted their therapeutic effects on RA by down-regulating the phosphorylation levels of PI3K, IKK, NF-κB and AKT.

TNF-α, IL-1β, and IL-6 play an important role in the development and progression of RA. TNF-α induces endothelial cells to express adhesion molecules, promoting leukocyte adhesion and infiltration of the vascular endothelium. This leads to local inflammation, and it also directly stimulates collagenase synthesis in articular chondrocytes, promoting synovial inflammation (Abrahams et al., 2000; Vizcarra, 2003). An increase in IL-1β in the joint will exacerbate synovial inflammatory cell infiltration and promote the formation of vasospasm, ultimately leading to cartilage and bone destruction (Liu et al., 2020). The promotion of neutrophil migration and monocyte infiltration by IL-6 is an important mechanism in the pathogenesis of RA (Pandolfi et al., 2020). The results of the present study have shown that the expression levels of TNF-α, IL-1β, and IL-6 were significantly reduced in the serum of the high-dose P-A group, indicating that the anti-inflammatory effect of the P-A drug pair may be related to the inhibition of the production of pro-inflammatory factors.

In this study, UPLC-Q-TOF-MS/MS technology was used to comprehensively and accurately identify the active components in the P-A drug pair. Moreover, its mechanism of action in the treatment of RA was further explored by using network pharmacology and animal experiments. The results provide an important theoretical basis for the further development of P-A drug pair for the treatment of RA.

## 5 Conclusion

The P-A drug pair exhibited good therapeutic effects on RA rats. In this study, UPLC-Q-TOF-MS/MS was used to analyze and identify the key chemical components of the P-A drug pair, and network pharmacology to investigate the mechanism of action. The levels of serum TNF-a, IL-1β, and IL-6 were measured by enzyme linked immunosorbent assay (ELISA). The positive expression of p-PI3K, p-IKK, p-NF-κB, and p-AKT in the synovial tissue of the ankle joint was detected by immunohistochemical analysis. The expression of PI3K, IKK, and AKT and their phosphorylation levels were determined by western blot in each group of rats. It was speculated that components, such as caffeic acid, quercetin, paeoniflorin, and baicalein, might act on key targets, such as PIK3R1, PIK3CA, AKT1, HSP90AA1, and IKBKB, to downregulate the phosphorylation levels of PI3K, IKK, and AKT. Furthermore, the PI3K/AKT/NF-κB signaling pathway was regulated by inhibiting the release of inflammatory mediators TNF-α, IL-1β, and IL-6.

Through the comprehensive analysis of network pharmacology and pharmacodynamics, this study preliminarily interpreted the effect and mechanism of P-A drug pair in the treatment of RA, and provided reference for the study of the effect and mechanism of Chinese herbal medicine in the treatment of RA. However, this study has some limitations. For example, the docking results should be verified by experiments such as Isothermal Titration Calorimetry and Surface Plasmon Resonance, and the P-A drug pair and its potential active components, targets and signaling pathways for the treatment of RA have not been fully studied and verified by experimental pharmacology (*in vitro* or *in vivo*). In the following, we will carry out these studies, and we will also adopt cutting-edge methods and technologies based on experimental pharmacology, network pharmacology and multi-omics and their comprehensive analysis to investigate the specific effects and mechanisms of P-A drug pair on RA.

## Data Availability

The datasets presented in this study can be found in online repositories. The names of the repository/repositories and accession number(s) can be found in the article/Supplementary Material.

## Abbreviations

P-A, Paeoniae radix rubra–Angelicae sinensis radix; RA, rheumatoid arthritis; ELISA, enzyme-linked immunosorbent assay; HE, hematoxylin-eosin; CIA, collagen-induced arthritis; UPLC-Q-TOF-MS/MS, Ultra-performance liquid chromatography–quadrupole-time of flight-mass spectrometry; PPI, protein–protein interaction; GO, gene ontology; KEGG, kyoto encyclopedia of genes and genomes; BPs, biological processes; CCs, cell compositions; MFs, molecular functions.
