# Supplementary material for: The mechanism of action of paeoniae radix rubra–angelicae sinensis radix drug pair in the treatment of rheumatoid arthritis through PI3K/AKT/NF-κB signaling pathway
Source: Front Pharmacol. 2023 Mar 13;14:1113810. doi: 10.3389/fphar.2023.1113810 (PMC10040578; doi:10.3389/fphar.2023.1113810)
Supplement: Supplementary file 1 [file DataSheet1.PDF]

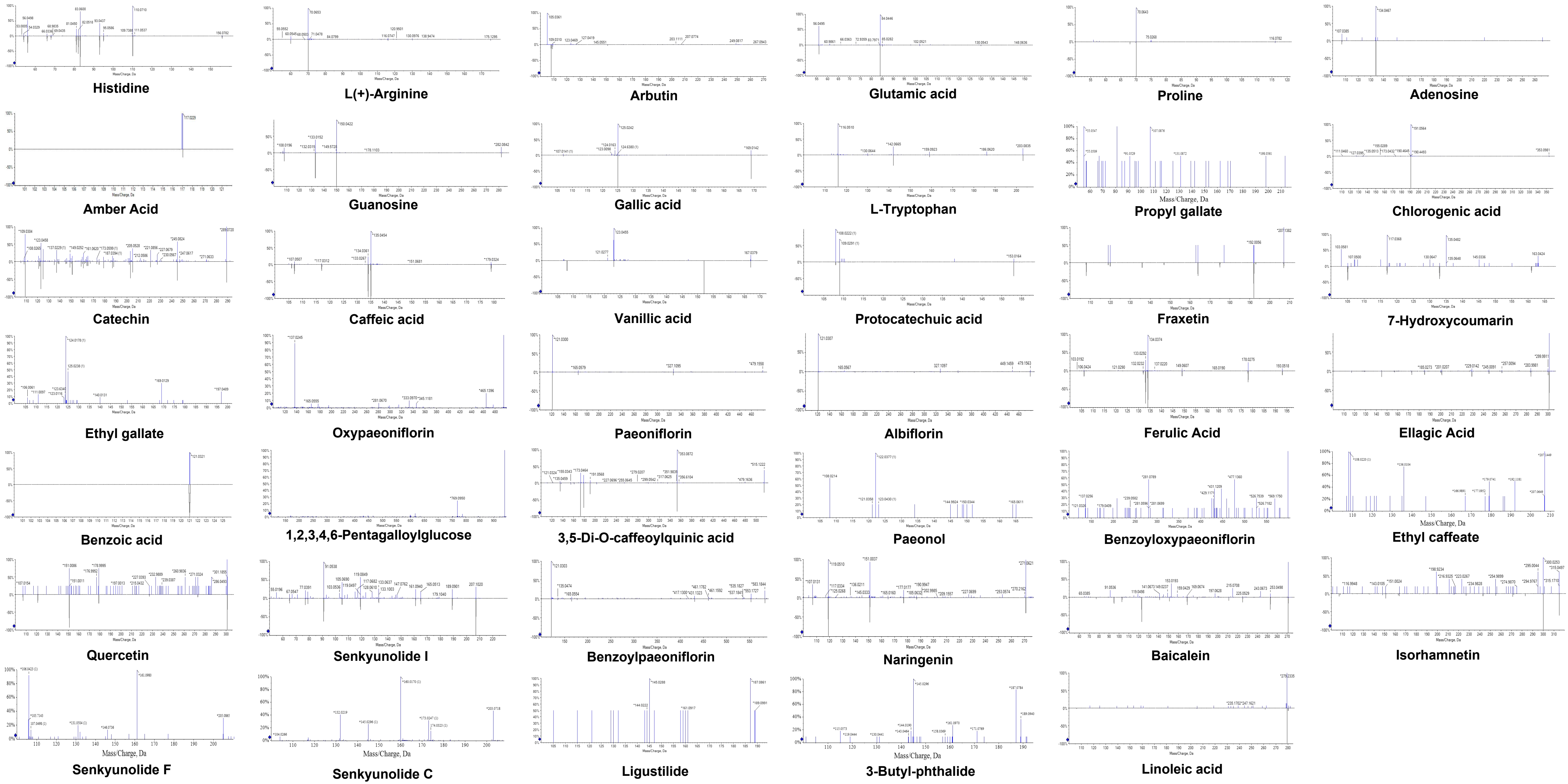
Histidine
L(+)-Arginine
Arbutin
Glutamic acid
Proline
Adenosine
Amber Acid
Guanosine
Gallic acid
L-Tryptophan
Propyl gallate
Chlorogenic acid
Catechin
Caffeic acid
Vanillic acid
Protocatechuic acid
Fraxetin
7-Hydroxycoumarin
Ethyl gallate
Oxypaeoniflorin
Paeoniflorin
Albiflorin
Ferulic Acid
Ellagic Acid
Benzoic acid
1,2,3,4,6-Pentagalloylglucose
3,5-Di-O-caffeoylquinic acid
Paeonol
Benzoyloxypaeoniflorin
Ethyl caffeate
Quercetin
Senkyunolide I
Benzoylpaeoniflorin
Naringenin
Baicalein
Isorhamnetin
Senkyunolide F
Senkyunolide C
Ligustilide
3-Butyl-phthalide
Linoleic acid
